# Salinity Stress Tolerance in Potato Cultivars: Evidence from Physiological and Biochemical Traits

**DOI:** 10.3390/plants11141842

**Published:** 2022-07-14

**Authors:** Satish Kumar Sanwal, Parveen Kumar, Hari Kesh, Vijai Kishor Gupta, Arvind Kumar, Ashwani Kumar, Babu Lal Meena, Giuseppe Colla, Mariateresa Cardarelli, Pradeep Kumar

**Affiliations:** 1 ICAR—Central Soil Salinity Research Institute, Karnal 132001, India; parveen.kumar@icar.gov.in (P.K.); harikeshkaul55@gmail.com (H.K.); arvind.kumar2@icar.gov.in (A.K.); ashwani.kumar1@icar.gov.in (A.K.); bl.meena@icar.gov.in (B.L.M.); 2 ICAR—Central Coastal Agricultural Research Institute, Ela, Old Goa 403402, India; 3 ICAR—Central Potato Research Institute, Regional Station Modipuram, Meerut 250110, India; vijay.gupta@icar.gov.in; 4 Department of Agriculture and Forest Sciences, University of Tuscia, 01100 Viterbo, Italy; giucolla@unitus.it; 5 ICAR—Central Arid Zone Research Institute, Jodhpur 342003, India; pradeep.kumar4@icar.gov.in

**Keywords:** *Solanum tuberosum*, salinity stress, antioxidant enzymes, oxidative stress

## Abstract

Salinity stress is a major constraint to sustainable crop production due to its adverse impact on crop growth, physiology, and productivity. As potato is the fourth most important staple food crop, enhancing its productivity is necessary to ensure food security for the ever-increasing population. Identification and cultivation of salt-tolerant potato genotypes are imperative mitigating strategies to cope with stress conditions. For this purpose, fifty-three varieties of potato were screened under control and salt stress conditions for growth and yield-related traits during 2020. Salt stress caused a mean reduction of 14.49%, 8.88%, and 38.75% in plant height, stem numbers, and tuber yield, respectively in comparison to control. Based on percent yield reduction, the genotypes were classified as salt-tolerant (seven genotypes), moderately tolerant (thirty-seven genotypes), and salt-sensitive genotypes (nine genotypes). Seven salt-tolerant and nine salt-sensitive genotypes were further evaluated to study their responses to salinity on targeted physiological, biochemical, and ionic traits during 2021. Salt stress significantly reduced the relative water content (RWC), membrane stability index (MSI), photosynthesis rate (Pn), transpiration rate (E), stomatal conductance, and K^+^/Na^+^ ratio in all the sixteen genotypes; however, this reduction was more pronounced in salt-sensitive genotypes compared to salt-tolerant ones. The better performance of salt-tolerant genotypes under salt stress was due to the strong antioxidant defense system as evidenced by greater activity of super oxide dismutase (SOD), peroxidase (POX), catalase (CAT), and ascorbate peroxidase (APX) and better osmotic adjustment (accumulation of proline). The stepwise regression approach identified plant height, stem numbers, relative water content, proline content, H_2_O_2_, POX, tuber K^+^/Na^+^, and membrane stability index as predominant traits for tuber yield, suggesting their significant role in alleviating salt stress. The identified salt-tolerant genotypes could be used in hybridization programs for the development of new high-yielding and salt-tolerant breeding lines. Further, these genotypes can be used to understand the genetic and molecular mechanism of salt tolerance in potato.

## 1. Introduction

Salinity stress is one of the major abiotic stresses which adversely affect plant growth and productivity. In India, about 6.73 million ha of land (~2.1% area of the country) is salt affected, out of which 2.96 million ha is saline and 3.77 million ha is sodic [1]. About 75% of salt-affected areas exist in four states, i.e., Gujarat, Maharashtra, West Bengal, and Rajasthan [2]. Scientific projections indicate that the salt-affected area in India is to be further increased at the pace of around 10% every year and by 2050, 16.2 million ha could be salt affected if preventive measures are not adopted [3], consequently affecting food and nutritional security. High salinity causes osmotic stress, nutrient stress, ion-specific toxicity, and water loss [4,5]. Salinity-induced oxidative stress leads to the accumulation of reactive oxygen species (ROS) such as singlet oxygen, superoxides, and hydroxy radicals, which reduce the membrane stability by causing lipid peroxidation that ultimately damages the cell membrane and alters normal cell functioning [6]. To minimize the negative effects of salinity stress, plants showed tolerance via different mechanisms such as maintaining turgor through osmolytes accumulation, ion exclusion from shoot and leaves, tissue tolerance, and shoot ion independent tolerance [7].

Potato is one of the most important staple food crops produced and consumed globally after rice, wheat, and maize [8]. It is a cheap and rich source of carbohydrates, proteins, vitamins, minerals, dietary fibers, and antioxidants, and plays an important role in global food security [9,10]. Potato crop is mainly grown for its tuber yield. Synthesis of sucrose through photosynthesis in leaves, its translocation, and conversion to starch in the stolon is an important physiological process for tuber formation and growth. However, any stress, during the tuber initiation process, negatively affects tuber yields and their quality [11]. Potato crop is moderately sensitive to salt stress because the threshold value of saturated soil extract (EC_e_) and irrigation water salinity (EC_iw_) is 1.7 dS m^−1^ and 1.1 dS m^−1^, respectively [12]. Salt stress at the reproductive stage negatively affects the photosynthesis process by disturbing the K^+^/Na^+^ ratio in leaves [13]. This abnormal K^+^/Na^+^ ratio decreases the movement of carbohydrates from source to sink and reduces the number and size of tubers [14]. Up to 60% yield reduction was recorded due to inhibition in the tuber formation process [15]. However, the magnitude of yield reduction due to salinity depends on duration, severity, and growth stage [16]. Salt stress during the initial stage is more harmful due to the reduced carbon assimilation rate and assimilates partitioning to the tubers [17]. Salt stress at toxic levels causes a reduction in the number of leaves, leaf water potential, chlorophyll content, stem number, dry matter, and concentrations of K^+^ in roots and stems [18,19] that ultimately hampers the tuber yield. 

It has been projected that potato yield will decrease by 18–32% due to various biotic and abiotic stresses during 2040–2069 [20]. Thus, this challenging situation necessitates the identification of potato genotypes that could tolerate stress conditions as well as best production practices to improve potato yield [11]. The present study assesses the production potential of fifty-three potato varieties under salt stress and unravels the responses of selected salt-tolerant and salt-sensitive genotypes to salinity on physiological, biochemical, and ionic traits.

## 2. Materials and Methods

### 2.1. Screening and Evaluation of Potato Genotypes under Saline Condition

A total of 53 diverse potato genotypes, collected from ICAR-Central Potato Research Institute, Regional Station, Modipuram, Meerut, India (Table 1), were planted in three replications under control and saline environments (EC_iw_ 6 dS m^−1^) in a randomized complete block design (RCBD) in last week of October 2020. Five sprouted tubers of size 35–40 mm of each variety were planted at a distance of 60 cm × 30 cm in Nain Experimental Saline Farm of the ICAR-Central Soil Salinity Research Institute situated at Panipat (Haryana), India. The initial and final soil salinity status of both control and saline treatments is given in Table 2. Salinity stress was created by applying saline irrigation water. Nain experimental farm area has natural saline ground water (EC_iw_ ~ 18 dS m^−1^) which was used to prepare the saline water of desired salinity (EC_iw_ 6 dS m^−1^) by diluting with good quality water, while for the control treatment, the best available water of EC_iw_ ~ 1.2 dS m^−1^ was used. Nain farm saline ground water has neutral pH with a dominance of Na^+^, Ca^2+^, Mg^2+^, Cl^−^, and SO_4_^2−^ ions. The treatment-wise irrigation was started just after planting and a total of 13 irrigations were applied during the whole cropping period on the basis of 100% evapotranspiration (ET). As per standard recommendation, half a dose of nitrogen and a full dose of phosphorus and potassium were applied at the time of planting and the remaining dose of nitrogen was applied at the earthing up stage (30 days after planting). The data were recorded on plant emergence, stem number, plant height, grade-wise tuber yield, and total tuber yield/plant. Dehaulming was performed 100 days after planting and harvesting was performed after one week to ensure proper curing. Treatment-wise soil samples were collected just after harvesting to measure the soil salinity build-up.

### 2.2. Morphological and Yield Traits

The observations for the plant height (cm) and no. of stems/plant of the three randomly selected and tagged plants from each replicate were measured at the time of physiological maturity (90 days after planting, DAP). Data of tuber yield were measured from three plants per replicate and grading was performed on the basis of tuber wt. (g), i.e., ≤20 g, 21–50 g, 51–75 g, and >75 g. Tuber dry weight was calculated from a known sample to a constant weight at 60 °C for 72 h and converted into percent.

On the basis of the screening experiment, sixteen contrasting genotypes were selected on percent yield reduction under saline conditions. Out of sixteen contrasting genotypes, seven genotypes had ≤25% yield reduction and were categorized as salinity tolerant, while nine genotypes had ≥50% yield reduction and were considered salinity sensitive. These 16 genotypes were again planted in microplots (size 3 m × 2 m) in October 2021 under control and saline conditions (EC_iw_ 6 dS m^−1^) for detailed analysis of physiological, biochemical, antioxidant enzymes, and ionic traits. The initial and final soil status of these microplots is presented in Table 2. Treatment-wise irrigation was applied just after planting and further irrigation was scheduled on the basis of 100% ET. 

### 2.3. Physiological Traits in Selected Contrasting Genotypes

All the physiological and biochemical traits were determined at 70 DAP. The relative water content (RWC) was measured in detached third and fourth fully expanded leaves from the top [21].

RWC = (FW − DW)/(FW − TW) × 100

where FW is the leaf fresh weight, DW is the leaf dry weight, and TW is the turgid leaf weight.

Leakage percentage of electrolyte was used to estimate the membrane thermo stability index in the leaves by the procedure of [22]. In 3rd fully expanded leaves, photosynthesis rate (Pn), stomatal conductance (gS), and transpiration rate (E) were estimated between 10:00 a.m. and 12:00 p.m. by using a portable photosynthetic system (Li 6800, Li-Cor Biosciences, Lincoln, NE, USA). Cuvette conditions were maintained at a photosynthetic photon flux density (PPFD) of 1000 µmol m^−2^ s^−1^, relative humidity > 60%, ambient CO_2_ concentration of 400 ppm, and leaf temperature of 25 °C [23]. Instantaneous water use efficiency (WUE) was also calculated as photosynthetic rate/transpiration rate (Pn/E) and intrinsic water use efficiency was calculated as photosynthetic rate/stomatal conductance (Pn/gS).

### 2.4. Biochemical Traits in Selected Contrasting Genotypes

Proline content of fresh leaves was determined by using ninhydrin reagent [24] and was quantified as mg g^−1^ fresh weight. Loreto and Velikova’s [25] approach was used to calculate H_2_O_2_ content. Fresh leaf tissue (0.3 g) was homogenized in 5% TCA and centrifuged for 15 min at 12,000× *g*. The supernatant was treated with 0.5 M potassium-phosphate buffer (pH 7.0) and 1 M potassium iodide (KI). The H_2_O_2_ concentration was represented as nmol g^−1^ FW and the absorbance was measured at 390 nm. The same supernatant used for H_2_O_2_ concentration was used to measure MDA content at 532 and 600 nm [26]. According to the modified approach, antioxidant enzymes, superoxide dismutase (SOD), and ascorbate peroxidase (APX) were extracted from leaves in a 0.1 M phosphate buffer (pH 7.5) containing 5% (*w*/*v*) polyvinylpolypyrrolidone, 1 mM EDTA, and 10 mM -mercapto-ethanol [27]. POX was extracted using 3 percent (*w*/*v*) polyvinylpolypyrrolidone in a 0.01 M phosphate buffer (pH 7.5). The capacity of the SOD enzyme to prevent the light-induced conversion of nitroblue tetrazolium (NBT) to formazan was measured [28]. One unit of APX corresponded to a change in O.D. of 1.0 per minute [29]. The POX activity was determined by using 1.0 mol of H_2_O_2_per minute [30]. Based on the breakdown of H_2_O_2_ at 240 nm, the catalase (CAT) activity was measured for 1 min [31].

### 2.5. Ionic Content in Selected Contrasting Genotypes

Na^+^ and K^+^ contents of leaves, roots, and tubers were determined at the harvest stage. Properly oven-dried and finely ground samples were digested in a 3:1 di-acid mixture (HNO_3_:H_2_SO_4_) for estimation of Na^+^ and K^+^ contents using a Flame Photometer 128 (Systronics India Ltd., Ahmedabad, India).

### 2.6. Statistical Analysis

In the first experiment, four observations were recorded (plant height, number of stems/plant, tuber yield/plant (g), and dry matter %) for preliminary screening under both environments. Two-way ANOVA was applied for estimating the effect of treatment, cultivar, and their interaction. Based on percent yield reduction under salinity stress, 7 tolerant (≤25%) and 9 susceptible (≥50%) potato cultivars were selected for the second experiment to reveal the physiological and biochemical basis of salinity tolerance.

In the second experiment, the distribution of each recorded parameter was tested through Shapiro–Wilk and Levene tests to comply with the homoscedasticity and assumptions of normality of residuals. Violated parameters were transformed using an appropriate transformation method. Further, to determine the effects of treatment, cultivars, and their interaction, two-way ANOVA was applied using STAR statistical software. The relative contribution of the physiological and biochemical traits was estimated based on the method proposed by Singh [32] to quantify genetic divergence in potato. To determine the significant differences in responses to salinity stress among cultivars, LSD tests were performed through the open-access online available statistical platform OPSTAT [33]. Pearson correlation coefficients were estimated to determine the association between morphological, physiological, and biochemical traits and salinity stress and control environment. All possible regression approaches were applied to prioritize the physico-biochemical traits, which were significantly associated (*p* ≤ 0.005) with tuber yield in potato. For ranking the potato cultivars under salinity stress, associated traits were considered in traits modeling (stepwise regression approach) to derive the response equation using statistical software STAR version 2.0.1 [34].

## 3. Results

### 3.1. Differential Genotypic Behaviour under Salinity Stress

All the genotypes differed significantly for all the studied parameters under control as well as stress conditions (Table 3). However, G × E interaction was non-significant for plant height, stem number/plant, yield/plant, and membrane stability index. Among these genotypes, plant height ranged from 18 cm (K. Naveen) to 50.57 cm (K. Chipsona-1) and from 16 cm (K. Khasigaro) to 47.80 cm (K. Chipsona-1), while stem numbers ranged from 2 (K. Lima) to 7.33 (K. Himsona) and from 1.93 (K. Lauvkar) to 6.87 (K. Himsona) under control and salt stress conditions, respectively (Table S1). The tuber yield per plant ranged from 120.80 g (K. Sheetman) to 559.33 g (K. Ganga) under control and from 60.93 g (K. Sheetman) to 428.27 g (K. Lalit) under salinity, while percent dry matter varied from 15.30 (K. Mohan) to 22.86 (K. Chipsona-3) and from 15.02 (K. Ganga) to 23.62 (K. Himsona) under control and stress conditions, respectively (Table S1). Salinity stress significantly reduced plant height, stem numbers, and tuber yield per plant to the tune of 14.49%, 8.88%, and 38.75%, respectively, in comparison to control (Table S2). The percent reduction under salinity stress relative to control varied significantly. Plant height reduced to maximum in K. Sutlej (32.60%) followed by K. Ashoka (30.49%) and K. Alankar (30.45%) while a minimum decline was in K. Naveen (1.11%), K. Surya (2.52%), and K. Neela (2.93%). Similarly, the decrease in stem number was maximum in K. Jeevan (42.78%) and K. Thar-3 (33.25%) whereas minimum in K. Jyoti (1.06%) and K. Neela (1.92%).

For tuber yield, salinity stress causes the highest reduction in K. Sangam (56.07%), K. Arun (55.61%), and K. Ganga (55.03%) while the lowest decline was observed in K. Thar-2 (7.15%), K. Giriraj (7.81%), and K. Lalit (21.21%) (Table S1). Based on percent tuber yield reduction due to salinity, genotypes were classified into three categories: salt-tolerant (<25% yield reduction), moderately tolerant (25–50% yield reduction), and salt-sensitive (>50% yield reduction). The seven tolerant genotypes were identified as K. Thar-2, K. Giriraj, K. Lalit, K. Surya, K. Jawahar, K. Neelkanth, and K. Red; nine sensitive genotypes were K. Manik, K. Kanchan, K. Alankar, K. Jeevan, K. Mohan, K. Sindhuri, K. Ganga, K. Arun, and K. Sangam; and the remaining thirty-seven genotypes were identified as moderately tolerant (Table 4). A further sixteen genotypes (7 tolerant and 9 sensitive) were used to verify the accuracy of screening for salt tolerance.

### 3.2. Comparative Response of Contrasting (Tolerant and Sensitive) Genotypes under Salinity Stress

#### 3.2.1. Physiological Stress Parameters

**Figure 1:**
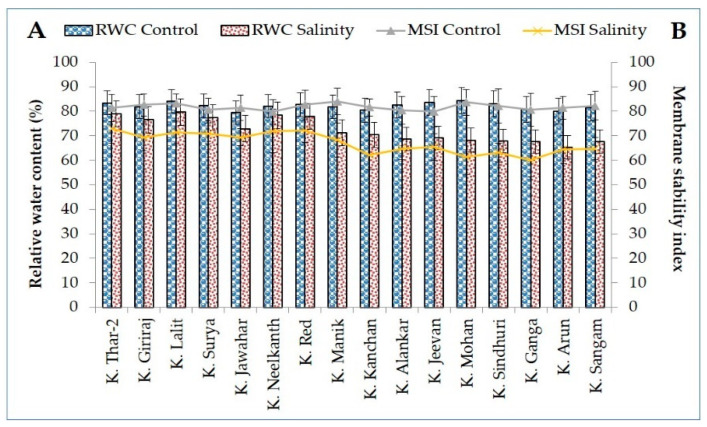
Relative water content (%), (**A**); and membrane stability index (%), (**B**) of tolerant and sensitive genotypes under control and salinity stress.

**Figure 2:**
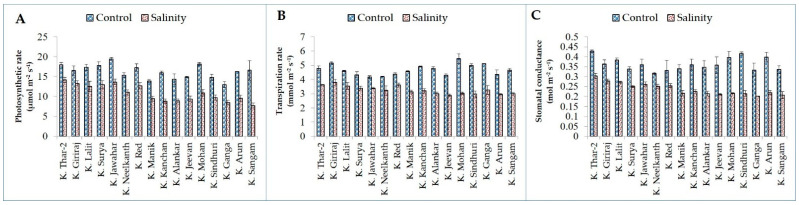
Gas exchange traits (photosynthetic rate, (**A**); transpiration rate, (**B**); and stomatal conductance, (**C**)) of tolerant and sensitive genotypes under control and salinity stress.

Salinity stress significantly reduced relative water content (RWC), membrane stability index (MSI), photosynthesis rate (Pn), transpiration (E), and stomatal conductance (gS) in both tolerant and susceptible potato genotypes (Figure 1 and Figure 2).

However, percent reduction was greater in salt-sensitive genotypes compared to salt-tolerant genotypes for all the examined parameters. The reduction for RWC was 5.68 (K. Arun) to 13.22% (K. Sangam) in salt-sensitive and 2.00 (K. Jawahar) to 6.47% (K. Giriraj) in tolerant genotypes. Likewise, about 13% reductions in membrane stability index were noticed in seven salt-tolerant genotypes, whereas ~22% decreases were found in nine salt-sensitive genotypes (Figure 1). Reduction in membrane stability index ranged from 9.87 (K. Neelkanth) to 15.98% (K. Giriraj) in salt-tolerant and from 18.15 (K. Jeevan) to 26.79% (K. Mohan) in salt-sensitive genotypes.

**Figure 3:**
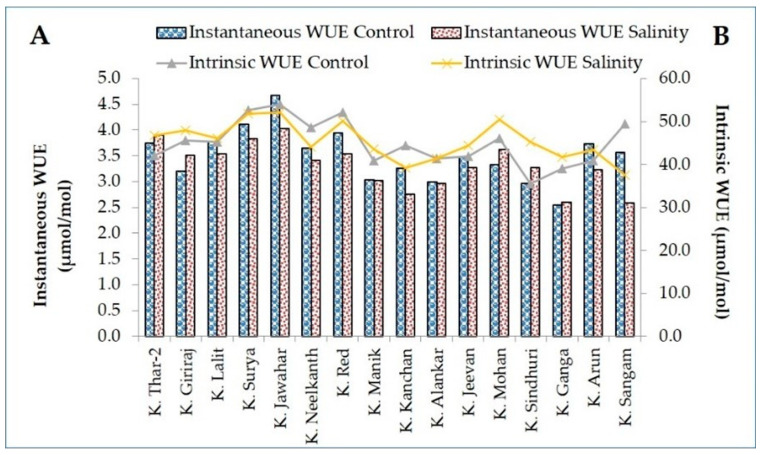
Water use efficiency [instantaneous water use efficiency (Pn/E), (**A**); and intrinsic water use efficiency (Pn/gS), (**B**)] of tolerant and sensitive genotypes under control and salinity stress.

Moreover, a maximum decline in photosynthesis rate, transpiration, and stomatal conductance was found in salt-sensitive genotypes K. Sangam (53.37%) and K. Kanchan (44.97%); K. Mohan (44.79%) and K. Sindhuri (40.28%); and K. Sindhuri (47.62%), K. Mohan (45%), and K. Arun (45%) while a minimum decrease was observed in salt-tolerant genotypes K. Giriraj (19.47%) and K. Thar-2 (21.15%); K. Red (18%) and K. Jawahar (18.27%); and K. Neelkanth (21.88%) and K. Giriraj (22.22%), respectively (Figure 2). Most of the genotypes showed a decrease in water use efficiency (WUE) (instantaneous) due to salt stress compared to control. However, water use efficiency (intrinsic) depicted a reverse trend. While comparing the percent reduction value, the maximum reduction was in salt-sensitive genotypes, i.e., 27.32% and 25.09% in K. Sangam for both intrinsic and instantaneous water use efficiency. Whereas a maximum reduction in salt-tolerant genotypes was found in K. Jawahar (13.70%) and K. Nelkanth (9.25%) for intrinsic and instantaneous water use efficiency, respectively (Figure 3). 

#### 3.2.2. Biochemical Stress Parameters

**Figure 4:**
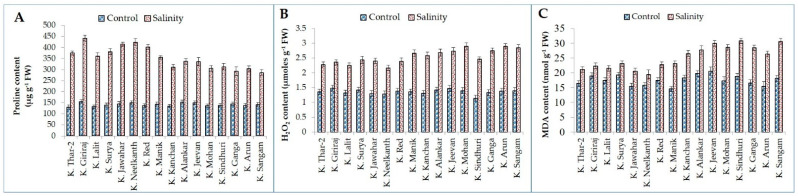
Biochemical parameters (proline, (**A**); H_2_O_2_, (**B**); and MDA, (**C**)) of tolerant and sensitive genotypes under control and salinity stress.

Imposition of salt stress significantly enhanced proline, malondialdehyde, and H_2_O_2_ contents in all potato genotypes. However, genotypic differences were observed for all studied biochemical parameters among salt-tolerant and sensitive genotypes (Figure 4). The proline content was increased to the tune of 172.53 (K. Surya) to 194.73% (K. Red) in salt-tolerant and 101.38 (K. Ganga) to 146.87% (K. Manik) in sensitive genotypes compared to control (Figure 4). The maximum increment for proline content was observed in K. Red (2.95-fold) and minimum in K. Ganga (2.01-fold). The increased level of H_2_O_2_ accumulation was observed in all sixteen genotypes of potato. Contrastingly, a high production of H_2_O_2_ was recorded in salt-sensitive genotypes than in the tolerant ones. For instance, the H_2_O_2_ production was 1.9 (K. Jeevan and K. Alankar) to 2.6 (K. Sindhuri) times in salt-sensitive while it was increased by only 1.6 (K. Giriraj) to 1.9 (K. Lalit) times in salt-tolerant genotypes (Figure 4). Similarly, MDA content increased in all genotypes. However, the reduction was higher in salt-sensitive genotypes (21.23 to 71.12%) than in salt-tolerant (17.72 to 45.61%) genotypes. The genotype K. Jawahar (1.46-fold) accumulated the highest MDA content while K. Surya (1.20-fold), K. Jeevan (1.21-fold), K. Neelkanth (1.23-fold), and K. Lalit (1.23-fold) were the lowest in the ranking for MDA accumulation (Figure 4).

**Figure 5:**
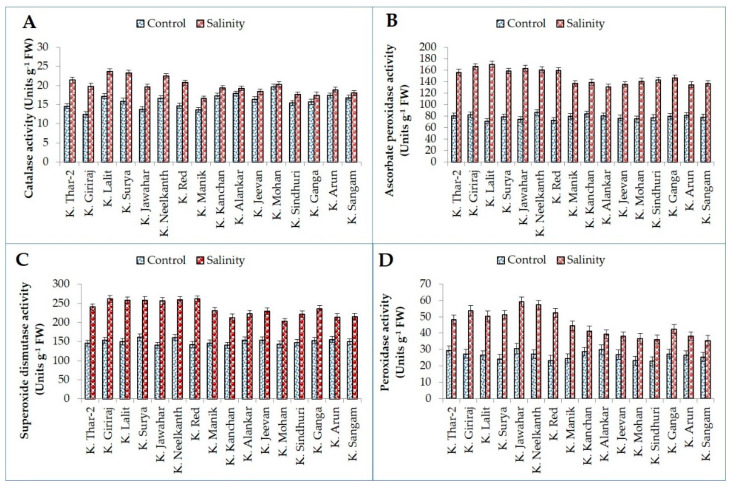
Antioxidative enzymes activity (CAT, (**A**); APX, (**B**); SOD, (**C**); and POX, (**D**)) of tolerant and sensitive genotypes under control and salinity stress.

The activities of antioxidant enzymes, catalase (CAT), ascorbate peroxidase (APX), superoxide dismutase (SOD), and peroxidase (POX) were increased significantly in all the genotypes under salinity stress. The salt-tolerant genotypes had a greater increase for all the enzymes than salt-sensitive genotypes (Figure 5). The CAT activity increased by 19.88 (K. Lalit) to 58.49% (K. Giriraj) in salt-tolerant and 3.15 (K. Mohan) to 22.16% (K. Manik) in salt-sensitive genotypes. Similarly, an increase in APX activity was fashioned for all potato genotypes due to salinity stress. APX activity was seen maximum in K. Lalit (170.40 units g^−1^ FW) followed by K. Giriraj (166.36 units g^−1^ FW) and K. Jawahar (163.46 units g^−1^ FW) while minimum in K. Alankar (130.54 units g^−1^ FW) followed by K. Arun (134.59 units g^−1^ FW) and K. Jeevan (135.30 units g^−1^ FW) in salt-stressed plants. APX activity ranged from 70.70 units g^−1^ FW in K. Lalit to 86.22 units g^−1^ FW in K. Neelkanth under control conditions (Figure 5). A similar pattern of greater SOD and POX activity was noticed in both tolerant and sensitive potato genotypes. The increased SOD and POX content was relatively high in K. Red (83.89% SOD; 126.25% POX) in salt-tolerant and in K. Manik (57.32% SOD; 80.89% POX) in salt-sensitive genotypes (Figure 5). The SOD activity was higher in K. Giriraj (261.48 units g^−1^ FW) and lower in K. Mohan (202.74 units g^−1^ FW) under salt stress conditions. The POX activity was highest in K. Jawahar (59.25 and 30.45 units g^−1^ FW) under both salt stress and control conditions while lowest in K. Sangam (35.46 units g^−1^ FW) under salt and in K. Sindhuri (22.90 units g^−1^ FW) under control condition (Figure 5).

#### 3.2.3. Ion Concentrations

The K^+^/Na^+^ ratio is an important trait to identify salt tolerance and it was noted that all sixteen potato genotypes in all the plant parts, i.e., root, leaf, and tubers, showed a gradual decrease under salt stress compared to control. However, this decline was observed maximum in salt-sensitive genotypes which was 38.21%, 50.48%, and 14.44% as compared to salt-tolerant genotypes 21.13%, 28.43%, and 8.38% under root, leaf, and tubers, respectively. Salt-sensitive genotypes K. Sangam (48.17%), K. Arun (60%), and K. Jeevan (24.15%) depicted maximum, whereas K. Manik (22.81%), K. Kanchan (29.86%), and K. Mohan (6.92%) exhibited minimum decline for root, leaf, and tubers, respectively (Table 5).

#### 3.2.4. Correlation Analysis

Tuber yield (TY) per plant was positively and significantly (*p* ≤ 0.001) correlated with plant height (PH), stem number (SN), relative water content (RWC), membrane stability index (MSI), and peroxidase (POX) under salt stress conditions; however, under control, it showed a significant positive association with plant height only (*p* ≤ 0.01). Stem number and membrane stability index had a positive but non-significant correlation with tuber yield and negative and moderate (*p* ≤ 0.01) correlation with tuber K^+^/Na^+^ ratio under control conditions (Table 6). Similarly, POX had a moderately significant (*p* ≤ 0.01) correlation with tuber yield under salt stress; however, no association was observed under control conditions. Proline (PRO) content showed a positive and significant (*p* ≤ 0.01) correlation with hydrogen peroxide (H_2_O_2_) under control while a negative and significant (*p* ≤ 0.001) association was observed under salinity conditions. Membrane stability index had a significantly positive (*p* ≤ 0.001) association with plant height, relative water content, stem numbers, proline content, and POX under salt stress; and a significant (*p* ≤ 0.01) negative correlation was found with stem numbers and negative and non-significant with plant height, relative water content, proline content, and POX under control conditions (Table 6). Tuber K^+^/Na^+^ showed a significant (*p* ≤ 0.01) and positive correlation with proline content and negative with stem number under control conditions. Under stressed conditions, tuber K^+^/Na^+^ ratio had a non-significant and weak positive association with relative water content, membrane stability index, proline content, and POX and a negative association with plant height stem number and H_2_O_2_.

#### 3.2.5. Genetic Divergence

Contribution of traits in total genetic divergence under salinity stress and the effect of salinity stress on various traits and direction of magnitude are given in Table 7. The sixteen genotypes varied greatly for percent alterations in different traits due to salinity stress. The maximum alteration was observed for proline content (148.61%) followed by ascorbate peroxidase (89.27%) and H_2_O_2_ (87.62%), while minimum alteration was noted in WUE (intrinsic; µmol/mol) (0.57%). That showed an increase in the mean value of these traits under salt stress compared to control. The plant height, stem numbers, tuber yield, relative water content, membrane stability index, photosynthesis rate, transpiration and stomatal conductance, water use efficiency (instantaneous), root K^+^/Na^+^, leaf K^+^/Na^+^, and tuber K^+^/Na^+^ showed alterations in the negative direction while the remaining traits showed alterations in the positive direction. The contribution of traits towards the total genetic diversity would help in the selection of divergent parents for their use in crop improvement programs. In the present study, the percent contribution of different traits revealed that relative water content (30.79%) followed by peroxidase (15.13%), membrane stability index (14.70%), ascorbate peroxidase (10.68%), superoxide dismutase (8.48%), tuber K^+^/Na^+^ (7.67%) malondialdehyde content (4.25%), and catalase (3.75%) collectively contributed more than 95% towards the genetic divergence while the remaining traits (4.57%) had a very small contribution.

#### 3.2.6. Potato Traits Priority under Salinity Stress

To determine the effect of component variables on tuber yield (dependent variable), all possible and stepwise regression analyses were performed. All possible regression analyses indicated that plant height, stem numbers, relative water content, proline content, H_2_O_2_, peroxidase, and tuber K^+^/Na^+^ ratio contributed significantly to tuber yield of potato under salt stress while the remaining traits, i.e., membrane stability index, root K^+^/Na^+^, and leaf K^+^/Na^+^ had a non-significant contribution to grain yield (Table S3). Therefore, these non-significant traits were removed during the stepwise regression approach. Results indicated that plant height, stem numbers, and POX collectively accounted for more than 70% of the total tuber yield variation under salt stress. Further, plant height, stem numbers, relative water content, H_2_O_2_, POX, tuber K^+^/Na^+^, and membrane stability index with cumulative R^2^ = 83.54 contributed significantly to tuber yield variation (Table 8) and could be best fitted since it reflected the smallest Mallows’ *Cp* criterion. Based on regression coefficients of respective traits, the following equation was computed for the estimation of predicted tuber yield under salt stress (Table S4).

Predicted tuber yield = −1643.69 + (12.47 × PH) + (40.82 × SN) + (18.36 × RWC) + (−0.83 × PRO) + (224.72 × H_2_O_2_) + (8.33 × POX) + (37.02 × Tuber K^+^/Na^+^) + (−11.41 × MI).

## 4. Discussion

Salt stress adversely affects the growth and developmental traits in potato by reducing root length, shoot length, plant height, number of branches, fresh and dry root weight, and plantlet weights which ultimately decreased the tuber yield of potato [35,36]. This decrease in plant growth and yield-related traits may occur due to excess accumulation of salts around the root zone that affects the water and nutrient uptake by the potato plantlets [37]. The tuber numbers, weight of tubers, and total tuber yield are also severely affected by salt stress causing a yield loss of up to 60% [15,38]. In the current study, a reduction in plant height, stem number, tuber yield, and dry matter was in the tune of 32.60%, 42.78%, 56.07%, and 10.42%, respectively, under salinity stress, although genotypic variations were observed. Based on percent tuber yield reduction under salt stress, seven genotypes were identified as salt-tolerant, thirty-seven as moderately tolerant, and nine as salt-sensitive genotypes, showing tuber yield reductions of less than 25%, 26% to 50%, and >50%, respectively. The selected tolerant and sensitive genotypes were re-evaluated in a further study to identify the biochemical, physiological, and ionic mechanisms of salinity tolerance. The better performance of salt-tolerant genotypes under stress conditions was found to be associated with maintenance of high K^+^/Na^+^ ratio, photosynthetic activity, accumulation of osmotic regulators, and higher activities of antioxidant enzymes that minimize the production of ROS [39,40,41]. The results of these studies were found consistent with our experimental findings.

Water plays an important role in the physiology of plants and maintaining higher leaf water content is the best strategy to mitigate salt stress [42,43]. Relative water content is a basic parameter that is commonly used as a physiological marker to screen out the tolerance for salinity stress. Our results showed that tolerant genotypes had a lower reduction in RWC compared to sensitive genotypes under salt stress, indicating their greater ability to pump out more water from the surroundings in the soil. A dramatic decrease in RWC under stress conditions was reported by many earlier researchers in different crops [44]. Additionally, all the physiological and biochemical processes such as stomatal closing and opening, photosynthesis, translocation of assimilate, and cell division are determined by the plant water status. Photosynthesis is an important source of energy for various metabolic activities in plants. Our data showed a significant reduction in photosynthetic rate, transpiration, and stomatal conductance of all the sixteen potato genotypes due to salinity stress. However, this decrease was more pronounced in salt-sensitive genotypes compared to control. The reduction in the photosynthetic rate due to salinity could be related to either stomatal factors or non-stomatal factors which ultimately determine the photosynthetic efficiency of the plant [45]. Generally, it is believed that stomatal closure is the earliest sign against any stress that reduces water uptake and lowers gaseous exchange under salt stress, which adversely affects photosynthesis, resulting in poor growth and tuber yield [46]. Similarly, a significant decline in stomatal conductance and CO_2_ assimilation was observed under 300 mM salt stress. Reduced stomatal conductance causes a reduction in intracellular CO_2_ and the activity of the RuBisco enzyme leads to decreased net photosynthetic rate [47]. Another possible reason might be a decrease in photosynthetic pigments due to enhanced chlorophyllase activity and reduced RuBPase activity also leading to a decline in the photosynthetic rate [48,49]. In addition, salinity stress adversely affects the availability of potassium which is an essential nutrient for the maintenance of the turgidity of guard cells. Therefore, disturbances in the turgidity of guard cells may be the possible reason for reduced transpiration and stomatal conductance under salt stress [50]. Similar to our findings, a significant reduction in net photosynthesis rate, transpiration rate, chlorophyll content, and stomatal conductivity was also reported in purslane, wheat, white willow, and eggplant [51,52,53,54], respectively.

The present findings revealed that water use efficiency, either instantaneous or intrinsic, decreased in all genotypes due to salt stress. However, the salt-tolerant genotypes were able to maintain normal homeostasis. These results are consistent with the findings of Levy et al. [55] and Wang et al. [56] in potato. The deposition of salts in the root zone decreases the osmotic potential which leads to a decrease in water potential that ultimately reduces water availability [15]. Membrane stability is another important parameter that is used to estimate the salt tolerance of different crops because relative water loss from plant cells negatively affects the membrane structure as well as its function [57]. In the present study, the tolerant genotypes displayed a relatively low reduction in MSI value in comparison to sensitive ones. Induction of ROS under stress conditions is correlated with electrolyte leakage and membrane damage [58]. Our results showed similarity with findings of [41,59,60] who reported less reduction in MSI in salt-tolerant genotypes.

The ionic imbalance due to salinity stress leads to the production of ROS that ultimately disrupt normal cell metabolism in potato plants [61]. The tolerant genotypes are able to avoid such damage by maintaining high K^+^/Na^+^ in the cytosol, accumulation of compatible solutes such as proline, and activation of antioxidant enzymes [62,63]. This was revealed by a higher percent increase in H_2_O_2_ and MDA production, which are the most commonly used biochemical markers for oxidative stress. From the results, it is clear that at the same salinity level, salt-sensitive genotypes accumulated more ROS while tolerant genotypes accumulated relatively less ROS. Previous studies showed that reduced H_2_O_2_, MDA content, and electrolyte leakage are useful biochemical traits to select tolerant genotypes against abiotic stress [64]. The greater activity of these ROS may be due to a less efficient antioxidant defense system and less osmolytes accumulation [65,66]. A positive association between increased antioxidant defense system and reduced oxidative stress was noticed under salt stress in many crops [67]. To examine the role of antioxidant enzymes such as SOD, APX, POX, and CAT against salinity stress, they were quantified to confirm the hypothesis. The experimental observations indicated that the activities of antioxidant enzymes increased in salt-tolerant genotypes compared to salt-sensitive ones. SOD is considered the most effective antioxidant enzyme in all aerobic organisms prone to oxidative stress due to its early defending role against abiotic stresses. Under salinity stress, superoxides are leaked from the electron transport chain of chloroplasts and mitochondria. SOD eliminates the superoxide ions by converting them to less toxic products H_2_O_2_ and oxygen, thus protecting potato cells from the toxic effect of salt stress [68,69]. The increased activity of SOD under abiotic stresses including salinity is found in many crop plants. H_2_O_2_ produced in the dismutation reaction by SOD is detoxified by both APX and CAT by converting it into water and oxygen; however, APX has more affinity for H_2_O_2_ than CAT, thus playing an essential role in ROS scavenging under stress [70,71]. Another adaptive response to stress conditions is the synthesis of higher proline content which plays an important role not only in osmotic adjustment but also in detoxification of ROS, membrane stability, and maintaining the structure and activity of other enzymes and proteins [72,73]. In our study, proline was observed as a valuable osmotic regulator for potato genotypes to survive under salt stress conditions. High proline production was found in salt-tolerant genotypes than sensitive genotypes. Consistent findings were reported in tomato [74] and okra [75] under salt stress. However, conflicting results were seen in different eggplant varieties; for instance, in some cases, tolerant genotypes accumulated more proline while in other studies sensitive genotypes produced more proline [76,77].

It is a well-known fact that Na^+^ and Cl^−^ accumulation increased in cell lines of potato under salinity stress and the K^+^/Na^+^ ratio was slightly higher in tolerant genotypes [78]. Higher accumulation of Na^+^ in roots maintains normal cell metabolism and restricts its transportation to leaves, therefore, limiting the Na^+^ accumulation in leaves [79]. In the present study, a significant decline in the K^+^/Na^+^ ratio was observed in all plant parts, i.e., roots, leaves, and tubers of sixteen genotypes, whereas leaves showed more decline than roots and tubers. Because Na^+^ and Cl^−^ are produced more in shoots than roots and leaves are more prone to Na^+^, this may be the probable reason that the K^+^/Na^+^ ratio is reduced more in leaves as compared to roots and tubers [80]. Similar to our findings, Kumar et al. [81] reported a higher concentration of Na^+^ and Cl^−^ ions in stem and leaves than in roots, defining that potato is not a better salt excluder crop. The higher ions in leaves lead to oxidative stress which adversely affects normal cell functioning. Further, a higher percent reduction was observed in salt-sensitive than salt-tolerant genotypes. Earlier studies in potato and pea also support our findings that more uptake of Na^+^ occurs in sensitive genotypes than salt-tolerant ones [82,83]. Salt-tolerant genotypes limit the accumulation of excess salt by their compartmentalization into different tissues mainly in vacuoles or through Na^+^ exclusion [84].

The analysis of variance showed significant differences for all the studied traits under control and salinity conditions indicating genetic differences among potato genotypes selected for salt tolerance, while the interaction was significant for most of the traits (Table 6). Our results showed a positive correlation of tuber yield with RWC, MSI, proline, and APX, indicating that osmotic adjustment and activation of antioxidant enzymes play very important roles in salt tolerance. Further, H_2_O_2_ correlated negatively with tuber yield, suggesting that low production of ROS during stress is good for high tuber yield in potato. A negative correlation of salt stress with plant growth and developmental traits was observed which essentially is due to the accumulation of toxic ions and deficiency of essential nutrients under salinity stress in wheat [85,86]. Regression analysis identified stem numbers, RWC, proline content, POX, and MSI as the most important traits contributing significantly toward the maximum variation for tuber yield in potato. Likewise, RWC, MSI, SOD, APX, POX, and CAT contributed more than 90% towards genetic diversity, indicating that consideration of these traits in the selection of divergent parents will be helpful in genetic improvement programs for potato. The stepwise regression approach in barley revealed that SOD and CAT collectively explained more than 95% of variation associated with the relative dry weight under salt stress [87]. Similarly, Ali et al. [88] reported that BADH activity in potato was positively determined by MDA, chlorophyll, and proline content. Taken together the overall results, the salt-tolerant genotypes were able to maintain relatively high RWC, MSI, antioxidants activity, and proline content than salt-sensitive genotypes.

## 5. Conclusions

The potato genotypes examined in the present study have a huge variation in measured morphological and yield traits. Under salinity stress, the identified tolerant genotypes confirm significantly diverse physiological and biochemical responses. The better performance of salt-tolerant genotypes under salt stress was due to the higher strong antioxidant defense system as evidenced by greater activity of superoxide dismutase (SOD), peroxidase (POX), catalase (CAT), and ascorbate peroxidase (APX) and better osmotic adjustment. The stepwise regression approach identified plant height, stem numbers, relative water content, proline content, H_2_O_2_, POX, tuber K^+^/Na^+^, and membrane stability index as predominant traits for tuber yield, suggesting their significant role in alleviating salt stress. The identified salt-tolerant genotypes can be recommended for cultivation under salinity stress and could be used in hybridization programs for the development of new high-yielding and salt-tolerant breeding lines. Further, these genotypes can be used to understand the genetic and molecular mechanisms of salt tolerance in potato.

## Figures and Tables

**Table 1:** Salient features of 53 potato cultivars.

Name	Parentage	Release Year	Maturity Group	Tuber Color	Tuber Shape	Flesh Color	Uses ^#^
K. Kundan *	Ekishirazu × Katahdin	1958	Medium	White	Round to ovoid	Cream	T
K. Safed	Clonal selection from Phulwa	1958	Late	White	Round	Light yellow	T
K. Red	Clonal selection from Darjeeling Red Round	1958	Medium	Red	Round	Yellow	T
K. Kuber	(*S. curtilobum* × *S. tuberosum*) × *S. andigenum*	1958	Medium	White	Ovoid	White	T
K. Kumar	Lumbri × Katahdin	1958	Late	White	Ovoid	White	T
K. Neela	Katahdin × Shamrock	1963	Late	White	Ovoid	White	T
K. Sindhuri	Kufri Red × Kufri Kundan	1967	Late	Red	Round	Cream	T
K. Jyoti	3069d (4) × 2814a (1)	1968	Medium	White	Ovoid	White	T
K. Jeevan	M 109-3 × Seedling 698-D	1968	Late	White	Ovoid	White	T
K. Chamatkar	Ekishirazu × Phulwa	1968	Late	White	Round	Yellow	T
K. Khasigaro	Taborky × Seedling 698-D	1968	Late	White	Round to ovoid	Cream	T
K. Sheetman	Craigs Defiance × Phulwa	1968	Medium	White	Ovoid	Cream	T
K. Alankar	Kennebec × ON 2090	1968	Medium	White	Ovoid	White	T
K. Naveen	3070d (4) × Seedling 692-D	1968	Late	White	Ovoid	White	T
K. Chandramukhi	Seedling 4485 × Kufri Kuber	1968	Early	White	Ovoid	White	T
K. Muthu	3046 (1) × M109-3	1971	Medium	White	Round to ovoid	White	T
K. Lauvkar	Serkov × Adina	1972	Early	White	Round	White	T
K. Badshah	Kufri Jyoti × Kufri Alankar	1979	Medium	White	Ovoid	White	T
K. Bahar	Kufri Red × Gineke	1980	Medium	White	Round to ovoid	White	T
K. Lalima	Kufri Red × AG 14 (Wis. × 37)	1982	Medium	Red	Round	White	T
K. Sherpa	Ultimus × Adina	1983	Medium	White	Round	Cream	T
K. Swarna	Kufri Jyoti × (VTn)2 62.33.3	1985	Medium	White	Round to ovoid	White	T
K. Megha	SLB/K-37 × SLB/Z-73	1989	Late	White	Round to ovoid	White	T
K. Ashoka	EM/C-1020 × Allerfruheste Gelbe	1996	Early	White	Ovoid	White	T
K. Sutlej	Kufri Bahar × Kufri Alankar	1996	Medium	White	Ovoid	White	T
K. Jawahar	Kufri Neelamani × Kufri Jyoti	1996	Medium	White	Round to ovoid	White	T
K. Chipsona-1	ME ×.750826 × MS/78-79	1998	Medium	White	Ovoid	White	C, F
K. Pukhraj	Craigs Defiance × JE×/B-687	1998	Early	White	Ovoid	Yellow	T
K. Giriraj	SLB/J-132 × E×/A 680-16	1998	Medium	White	Ovoid	White	T
K. Anand	Kufri Ashoka × PH/F-1430	1999	Medium	White	Ovoid to oblong	White	T
K. Kanchan	SLB/Z-405(a) × Pimpernel	1999	Medium	Red	Ovoid to oblong	Cream	T
K. Shailja	Kufri Jyoti × E×/A 680-16	2005	Medium	White	Round to ovoid	White	T
K. Pushkar	QB/A 9-120 × Spatz	2005	Medium	White	Round to ovoid	Light yellow	T
K. Arun	Kufri Lalima × MS/82-797	2005	Medium	Red	Ovoid	Cream	T
K. Chipsona-3	MP/91-86 × Kufri Chipsona-2	2006	Medium	White	Round to ovoid	Cream	C, F
K. Himalini	I-1062 × Tollocan	2006	Medium	White	Ovoid to oblong	Cream	T
K. Surya	Kufri Lauvkar × LT-1	2006	Early	Yellow	Oblong	Yellow	T
K. Lalit	85-P-670 × CP 3192	2007	Medium	Light red	Round	Light yellow	T
K. Himsona	MP/92-35 × Kufri Chipsona-2	2008	Medium	White	Round to ovoid	Cream	C
K. Sadabahar	MS/81-145 × PH/F-1545	2008	Medium	White	Oblong	White	T
K. Girdhari	Kufri Megha × Bulk pollen of 10 genotypes	2008	Medium	White	Ovoid to oblong	Pale yellow	T
K. Frysona	MP/92-30 × MP/90-94	2009	Medium	White	Long oblong	White	F
K. Chipsona-4	Atlantic × MP/92-35	2010	Medium	White	Round	White	C
K. Mohan	MS/92-1090 × CP 1704 (Claudia)	2015	Medium	White	Ovoid	White	T
K. Lima	C90.266 × C93.154	2018	Medium	Creamy white	Ovoid	Cream	T
K. Neelkanth	MS/89-1095 × CP 3290	2018	Medium	Purple	Ovoid	Yellow	T
K. Ganga	MS/82-638 × JX576	2018	Medium	Creamy white	Ovoid	Cream	T
K. Sangam	Kufri Himsona × Kufri Pukhraj	2020	Medium	Creamy white	Ovoid	White	T, C, F
K. Thar-3	JN 2207 × Kufri Jyoti	2020	Medium	White	Oval	Cream	T
K. Manik	Kufri Arun × CP3192	2020	Medium	Red	Round	Yellow	T
K. Thar-1	Kufri bahar × CP 1785	2020	Medium	Creamy white	Round to oval	Cream	T
K. Thar-2	CIP389468.3 × 88.052	2020	Medium	Light yellow	Ovoid	Light yellow	T
K. Fryom	Kufri Chipsona-1 × MP/92-35	2020	Medium	White	Oblong	White	F

* K. is abbreviated form of Kufri, used as prefix in each cultivar/genotype name; ^#^ Suitable for table purposes (T), chips (C), and French fries (F).

**Table 2:** Soil status: Initial and final soil salinity and alkalinity.

Parameters	Initial Soil Status	Final Soil Status	
		Control Treatment	Saline Treatment
2020			
ECe (dS m^−1^)	1.28	1.42	6.24
pHs	7.52	7.56	7.50
2021			
ECe (dS m^−1^)	1.36	1.54	6.46
pHs	7.81	7.78	7.88

**Table 3:** Variance analysis for the recorded traits in selected (16) potato genotypes under control and salinity treatment.

Variables	Mean Squares		F Values		Significance	
	Genotypes	G × E	Genotypes	G × E	Genotypes	G × E
Df	15	15	15	15	Pr (>F)	Pr (>F)
Plant height (cm)	96.05	12.52	4.10	0.53	0.000	0.911
Stem number (nos)	4.60	0.63	5.94	0.81	0.000	0.660
Yield/plant (g)	40,103.05	9613.05	3.87	0.93	0.000	0.539
RWC (%)	46.52	32.85	1058.77	747.68	0.000	0.000
MSI (%)	100.18	84.75	1.84	1.56	0.049	0.113
SPAD	70.40	17.54	3.66	0.91	0.000	0.556
Proline (µg g^−1^ FW)	4128.90	3507.76	53.74	45.66	0.000	0.000
H_2_O_2_ (µmoles g^−1^ FW)	0.11	0.08	110.15	78.74	0.000	0.000
MDA (nmol g^−1^ FW)	37.57	15.84	204.58	86.26	0.000	0.000
CAT (units g^−1^ FW)	13.29	9.69	310.72	226.45	0.000	0.000
APX (units g^−1^ FW)	247.10	322.85	581.65	759.98	0.000	0.000
SOD (units g^−1^ FW)	795.79	594.60	596.46	445.67	0.000	0.000
POX (units g^−1^ FW)	119.84	87.03	422.44	306.80	0.000	0.000
Pn (µmol CO_2_/m^2^/s)	19.32	3.25	12.64	2.13	0.000	0.020
E (mmol H_2_O/m^2^/s)	0.33	0.33	24.67	24.57	0.000	0.000
gS (mol H_2_O/m^2^/s)	0.01	0.00	9.84	8.58	0.000	0.000
WUE (instantaneous; µmol/mmol)	1.15	0.21	12.15	2.22	0.000	0.015
WUE (intrinsic; µmol/mol)	101.01	38.13	6.21	2.34	0.000	0.010
Root K^+^/Na^+^	0.31	0.34	31.38	33.83	0.000	0.000
Leaf K^+^/Na^+^	0.54	0.98	3.80	6.90	0.000	0.000
Tuber K^+^/Na^+^	4.27	0.56	57.95	7.57	0.000	0.000

**Table 4:** Grouping of potato genotypes based on % tuber yield reduction due to salinity stress.

**Salt-Tolerant Genotypes**	**Tuber Yield Reduction (<25%)**	**Moderately Tolerant Genotypes**	**Tuber Yield Reduction (25–50%)**	**Salt-Sensitive Genotypes**	**Tuber Yield Reduction (>50%)**
K. Thar-2	7.16	K. Megha	26.56	K. Manik	51.71
K. Giriraj	7.81	K. Sherpa	26.78	K. Kanchan	52.26
K. Lalit	21.21	K. Sadabahar	27.06	K. Alankar	53.73
K. Surya	23.06	K. Neela	28.01	K. Jeevan	53.92
K. Jawahar	23.80	K. Pushkar	28.58	K. Mohan	53.95
K. Neelkanth	23.88	K. Kundan	28.61	K. Sindhuri	54.97
K. Red	24.80	K. Lalima	28.67	K. Ganga	55.03
		K. Thar-1	28.87	K. Arun	55.62
		K. Chipsona-1	29.39	K. Sangam	56.07
		K. Chandramukhi	30.40		
		K. Kuber	31.53		
		K. Bahar	32.12		
		K. Swarna	32.81		
		K. Chipsona-3	35.18		
		K. Badshah	36.39		
		K. Fryom	37.92		
		K. Muthu	39.64		
		K. Thar-3	39.66		
		K. Jyoti	40.73		
		K. Chamatkar	40.96		
		K. Naveen	41.24		
		K. Chipsona-4	42.15		
		K. Shailja	43.18		
		K. Himsona	43.29		
		K. Frysona	44.22		
		K. Pukhraj	45.93		
		K. Lima	46.01		
		K. Girdhari	46.18		
		K. Ashoka	46.52		
		K. Anand	48.35		
		K. Himalini	48.60		
		K. Safed	48.71		
		K. Kumar	49.14		
		K. Sutlej	49.44		
		K. Sheetman	49.55		
		K. Lauvkar	49.72		
		K. Khasigaro	49.88		

**Table 5:** K^+^/Na^+^ ratio of tolerant and sensitive genotypes under control and salinity stress.

Varieties	Root K^+^/Na^+^		Leaf K^+^/Na^+^		Tuber K^+^/Na^+^	
	Control	Salinity	Control	Salinity	Control	Salinity
K. Thar-2	3.86 ± 0.01	2.68 ± 0.32	3.12 ± 0.99	3.08 ± 0.03	6.12 ± 0.01	5.49 ± 0.06
K. Giriraj	3.94 ± 0.01	2.86 ± 0.01	3.90 ± 0.01	2.79 ± 0.33	7.33 ± 0.01	5.40 ± 0.01
K. Lalit	3.65 ± 0.02	3.09 ± 0.01	3.65 ± 0.02	2.96 ± 0.03	4.05 ± 0.87	4.96 ± 0.01
K. Surya	3.43 ± 0.01	2.41 ± 0.01	4.28 ± 0.02	3.04 ± 0.03	5.65 ± 0.02	4.77 ± 0.03
K. Jawahar	3.98 ± 0.01	3.12 ± 0.03	4.65 ± 0.03	2.90 ± 0.06	6.54 ± 0.03	5.85 ± 0.03
K. Neelkanth	3.25 ± 0.03	3.18 ± 0.01	5.09 ± 0.03	2.98 ± 0.03	5.17 ± 0.03	4.97 ± 0.01
K. Red	3.56 ± 0.01	2.81 ± 0.01	4.82 ± 0.03	2.78 ± 0.01	4.14 ± 0.02	3.54 ± 0.03
K. Manik	3.42 ± 0.01	2.64 ± 0.03	4.96 ± 0.01	2.14 ± 0.03	4.72 ± 0.02	3.83 ± 0.03
K. Kanchan	3.80 ± 0.02	2.58 ± 0.01	3.45 ± 0.64	2.42 ± 0.01	4.21 ± 0.09	3.83 ± 0.01
K. Alankar	3.45 ± 0.02	2.12 ± 0.01	4.70 ± 0.01	2.18 ± 0.03	5.37 ± 0.03	4.42 ± 0.01
K. Jeevan	3.66 ± 0.02	2.25 ± 0.03	4.10 ± 0.02	2.32 ± 0.01	6.21 ± 0.05	4.71 ± 0.01
K. Mohan	3.45 ± 0.01	2.36 ± 0.03	3.98 ± 0.01	2.16 ± 0.03	6.50 ± 0.05	6.05 ± 0.09
K. Sindhuri	3.88 ± 0.01	2.08 ± 0.01	4.58 ± 0.04	2.12 ± 0.03	6.41 ± 0.01	5.39 ± 0.01
K. Ganga	3.68 ± 0.01	2.19 ± 0.01	4.31 ± 0.05	1.96 ± 0.03	5.26 ± 0.01	4.53 ± 0.03
K. Arun	3.71 ± 0.01	2.03 ± 0.01	4.80 ± 0.02	1.92 ± 0.01	4.54 ± 0.01	3.93 ± 0.03
K. Sangam	3.82 ± 0.01	1.98 ± 0.01	4.56 ± 0.02	1.98 ± 0.01	6.26 ± 0.02	5.63 ± 0.03
CV (%)	1.72	5.49	11.96	6.28	2.51	0.97
LSD (*p* ≤ 0.05)	0.11	0.23	0.86	0.26	0.23	0.08

**Table 6:** Correlation matrix among the selected traits under control (upper diagonal) and salinity stress condition (lower diagonal).

Variables	PH	SN	RWC	MSI	PRO	H_2_O_2_	POX	Tuber K^+^/Na^+^	TY
PH	0.558 ***	0.145	0.101	−0.065	−0.042	−0.035	−0.025	−0.196	0.415 **
SN	0.256	0.400 **	0.257	−0.385 **	0.021	−0.131	−0.091	−0.321 **	0.225
RWC	0.334 **	0.414 **	0.318 **	−0.127	−0.180	0.115	−0.386 **	0.094	−0.085
MSI	0.331 **	0.479 ***	0.899 ***	0.055	−0.263	−0.199	−0.069	−0.102	0.147
PRO	0.330 **	0.441 **	0.790 ***	0.811 ***	0.265	0.326 **	0.242	0.349 **	−0.073
H_2_O_2_	−0.305 **	−0.408 **	−0.892 ***	−0.796 ***	−0.782 ***	0.265	0.119	0.151	−0.246
POX	0.275 *	0.439 **	0.818 ***	0.789 ***	0.892 ***	−0.811 ***	0.283 *	0.065	−0.060
Tuber K^+^/Na^+^	−0.183	−0.198	0.060	0.012	0.093	−0.135	0.068	0.755 ***	−0.400 **
TY	0.770 ***	0.485 ***	0.536 ***	0.455 ***	0.451 ***	−0.449 ***	0.511 ***	0.010	0.545 ***

Significance *, **, *** at *p* ≤ 0.05, 0.01 and 0.001, respectively. Green color of diagonal values are indicating intra association of the trait in different environments (control and salinity). PH—plant height (cm), SN—number of stems/plant, RWC—relative water content, MSI—membrane stability index, PRO—proline, H_2_O_2_—hydrogen peroxide, POX—peroxidase, Tuber K^+^/Na^+^—K/Na ratio in tuber, TY—tuber yield.

**Table 7:** Relative contribution of different physiological, biochemical, and yield traits toward genetic divergence in potato genotypes.

Traits	Contribution (%)	Traits Mean ± SD		Alteration (%)	Direction of Magnitude
		Control	Salinity Stress		
Plant height (cm)	0.028	31.18 ± 5.66	26.58 ± 7.39	14.73	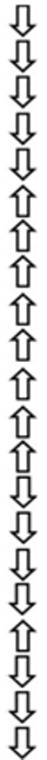
Stem Number (nos)	0.066	4.10 ± 1.29	3.48 ± 1.03	14.97	
Tuber Yield(g)	0.034	380.96 ± 131.66	232.64 ± 129.80	38.93	
RWC (%)	30.790	82.13 ± 1.43	72.35 ± 4.83	11.91	
MSI (%)	14.695	80.23 ± 10.75	67.00 ± 4.18	16.49	
SPAD	0.023	45.71 ± 5.34	47.51 ± 5.09	3.93	
Proline (µg g^−1^ FW)	0.761	141.64 ± 7.70	352.14 ± 49.84	148.61	
H_2_O_2_ (µmoles g^−1^ FW)	1.638	1.36 ± 0.08	2.55 ± 0.24	87.62	
MDA (nmol g^−1^ FW)	4.245	17.39 ± 1.76	25.17 ± 3.77	44.69	
CAT (units g^−1^ FW)	3.752	15.96 ± 1.80	19.80 ± 2.03	24.05	
APX (units g^−1^ FW)	10.681	78.56 ± 3.98	148.70 ± 12.90	89.27	
SOD (units g^−1^ FW)	8.477	149.63 ± 6.48	236.23 ± 20.09	57.88	
POX (units g^−1^ FW)	15.127	26.46 ± 2.39	45.29 ± 7.79	71.17	
Pn (µmol CO_2_/m^2^/s)	0.592	16.22 ± 2.18	10.83 ± 2.11	33.24	
E (mmol H_2_O/m^2^/s)	0.191	4.67 ± 0.38	3.25 ± 0.29	30.50	
gS (mol H_2_O/m^2^/s)	0.146	0.36 ± 0.04	0.24 ± 0.03	34.50	
WUE (instantaneous; µmol/mmol)	0.118	3.50 ± 0.58	3.32 ± 0.48	5.00	
WUE (intrinsic; µmol/mol)	0.223	45.09 ± 6.74	45.35 ± 4.54	0.57	
Root K^+^/Na^+^	0.394	3.66 ± 0.21	2.52 ± 0.41	31.03	
Leaf K^+^/Na^+^	0.356	4.31 ± 0.70	2.48 ± 0.44	42.40	
Tuber K^+^/Na^+^	7.666	5.53 ± 1.03	4.83 ± 0.76	12.62	

**Table 8:** Traits modeling for salinity tolerance though multiple linear regressions approach.

Dependent Variable	Step and Variables	C(p)	R-Square	Adj R-Square
TY (tubers yield)	1. PH	52.68	59.21	58.32
	2. PH + POX	31.79	68.87	67.48
	3. PH + SN + POX	25.23	72.48	70.60
	4. PH + SN + POX + Tuber K^+^/Na^+^	20.89	75.15	72.84
	5. PH + SN + PRO + POX + Tuber K^+^/Na^+^	14.93	78.51	75.95
	6. PH + SN + RWC + H_2_O_2_ + Tuber K^+^/Na^+^ + MSI	14.52	79.53	76.53
	7. PH + SN + RWC + H_2_O_2_ + POX + Tuber K^+^/Na^+^ + MSI	10.98	81.87	78.69
	8. PH + SN + RWC + PRO + H_2_O_2_ + POX + Tuber K^+^/Na^+^ + MSI	9.00	83.54	80.17

Mallows’ *Cp* Criterion is a way to assess the fit of a multiple regression model; smaller *Cp* values are better as they indicate smaller amounts of unexplained error.

## Data Availability

All the relevant data of the study is provided in the manuscript and supplementary files.

## Supplementary Materials

The following supporting information can be downloaded at: https://www.mdpi.com/article/10.3390/plants11141842/s1, Table S1: Basic statistics of plant height, number of stems/plant, and tuber yield/plant in 53 genotypes of potato under control and salinity stress condition. Table S2: Traits# (plant height, number of stems/plant, and tuber yield/plant) observations in 53 genotypes of potato under control and salinity stress condition. Table S3: Traits prioritization in salinity stress through stepwise regression that can be used for model predictions. Table S4: Regression coefficient, standard error, and significance of the prioritized traits for salinity stress tolerance.
