# Supplementary material for: Salinity Stress Tolerance in Potato Cultivars: Evidence from Physiological and Biochemical Traits
Source: Plants (Basel). 2022 Jul 14;11(14):1842. doi: 10.3390/plants11141842 (PMC9316722; doi:10.3390/plants11141842)
Supplement: Supplementary file 1 [file plants-11-01842-s001.zip › plants-1790453-supplementary.pdf]

Table S1. Traits# (plant height, number of stems/plant and tuber yield/plant) observations in 53 genotypes of potato under control and salinity stress condition

Genotype name*	Control			Salinity		
	Plant height (cm)	Stem numbers	Tuber Yield/ plant (g)	Plant height (cm)	Stem numbers	Tuber Yield/ plant (g)
K. Sangam	26.80	3.37	362.17	19.97	2.97	159.10
K. Thar-3	21.07	4.00	364.47	19.10	2.67	219.93
K. Chipsona-3	26.30	6.67	340.00	24.73	5.33	220.40
K. Frysona	32.00	2.93	278.73	25.17	3.40	155.47
K. Ashoka	24.93	4.33	299.60	17.33	4.03	160.23
K. Kundan	29.07	4.50	391.17	25.60	4.27	279.27
K. Sutlej	34.97	3.73	363.47	23.57	3.80	183.77
K. Jyoti	22.87	2.83	314.70	23.67	2.80	186.53
K. Shailja	31.77	3.60	337.60	26.33	3.80	191.83
K. Jeevan	33.13	5.47	248.87	23.10	3.13	114.67
K. Neela	23.90	3.13	261.07	23.20	3.07	187.93
K. Chamatkar	25.63	4.50	205.17	23.57	3.57	121.13
K. Manik	32.20	3.73	429.33	26.77	3.80	207.33
K. Thar-1	30.50	5.40	397.87	26.37	5.60	283.00
K. Himsona	25.03	7.33	273.67	21.20	6.87	155.20
K. Jawahar	24.60	3.73	341.67	22.77	3.33	260.33
K. Safed	36.40	5.50	425.63	26.20	4.90	218.33
K. Mohan	25.03	2.87	383.93	18.70	2.57	176.80
K. Chipsona-1	50.57	6.73	367.53	47.80	5.73	259.53
K. Megha	24.70	3.67	262.27	23.40	4.87	192.60
K. Khasigaro	18.13	3.40	245.10	16.00	2.57	120.83
K. Muthu	23.63	2.80	323.40	22.57	3.10	195.23
K. Sadabahar	34.53	2.60	381.03	29.27	2.73	277.93
K. Swarna	28.63	4.07	316.13	27.27	2.73	212.40
K. Girdhari	24.73	2.80	182.47	21.27	2.33	98.23
K. Badshah	40.10	3.47	486.80	35.40	3.87	309.67
K. Anand	34.23	3.27	349.13	26.57	3.00	180.33
K. Sheetman	24.93	4.50	120.80	18.87	3.70	60.93
K. Alankar	28.80	3.80	308.20	20.03	3.80	142.60
K. Pushkar	27.80	4.67	379.27	28.00	4.07	270.87
K. Lalima	27.73	2.60	246.47	25.30	3.20	175.80
K. Lima	36.60	2.00	492.93	33.20	2.00	266.13
K. Naveen	18.00	2.90	260.70	17.80	2.57	153.20
K. Sherpa	20.03	3.73	194.27	20.73	4.40	142.27
K. Chipsona-4	44.93	3.67	357.93	31.80	3.07	207.07
K. Neelkanth	28.37	5.93	473.67	29.77	5.73	360.53
K. Arun	34.77	3.67	405.00	30.57	3.27	179.77
K. Kanchan	32.13	3.27	393.20	26.30	2.37	187.73
K. Sindhuri	33.43	4.33	329.07	29.10	3.30	148.17

K. Red	36.00	5.53	442.00	32.10	4.93	332.40
K. Kuber	32.10	5.40	474.00	26.03	5.67	324.53
K. Thar-2	36.00	3.47	322.13	33.27	3.17	299.10
K. Bahar	30.90	2.60	420.93	25.00	3.57	285.73
K. Pukhraj	27.93	2.63	361.20	20.30	2.40	195.27
K. Lauvkar	29.17	2.07	303.33	22.77	1.93	146.50
K. Himalini	26.33	3.90	229.83	21.47	2.93	118.13
K. Kumar	28.27	3.60	352.33	21.07	3.27	179.20
K. Surya	26.57	3.73	237.07	25.90	2.73	182.40
K. Ganga	32.67	3.93	559.33	26.50	3.17	251.53
K. Chandramukhi	33.80	3.47	372.53	29.73	3.27	259.27
K. Giriraj	35.10	3.03	316.23	31.97	3.33	291.53
K. Fryom	36.30	4.40	383.83	34.13	3.40	238.27
K. Lalit	33.17	5.67	543.53	28.50	4.20	428.27

#-Values are the mean of three replicates *K-Kufri

Table S2. Basic statistics of of plant height, number of stems/plant and tuber yield/plant in 53 genotypes of potato under control and salinity stress condition

Variable	Min		Max		Mean		CV	
	**Control**	**Salinity**	**Control**	**Salinity**	**Control**	**Salinity**	**Control**	**Salinity**
Plant height (Cm)	17.98	16.01	50.58	47.80	25.61	29.95	20.72	21.73
Stem numbers (nos)	2.00	1.93	7.33	6.87	3.94	3.59	30.07	29.62
Tuber Yield/ plant (g)	120.80	60.94	559.33	428.27	343.64	210.47	26.17	34.17
Dry matter %	15.30	15.02	22.86	23.62	18.39	19.07	10.86	10.15

Table S3. Traits prioritization in salinity stress through stepwise regression that can be used for model predictions

Retained Variable	Regression coefficients (β_s)	Std. Error	t value	Pr(>|t|)
**Intercept**	-1782.63	489.01	-3.65	0.0008
**PH**	12.46	1.25	9.99	0.0000
**SN**	35.71	9.92	3.60	0.0009
**RWC**	20.19	6.19	3.26	0.0024
**PRO**	-0.89	0.41	-2.17	0.0363
**H_2O_2**	209.36	87.52	2.39	0.022
**POX**	7.35	3.08	2.38	0.0225
**Tuber K^+/Na^+**	39.54	12.00	3.29	0.0022
**MI**	-9.20	5.18	-1.77	0.0842
**Root K^+/Na^+**	54.88	40.61	1.35	0.1848
**Leaf K^+/Na^+**	-69.16	51.54	-1.34	0.1878
		**ANOVA**		
**Source**	**DF**	**Mean Square**	**F Value**	**Pr(> F)**
**Model**	10	67461.2539	21.28	0.0000
**Error**	37	3170.1191		
**Root MSE**	56.30			
**CV(%)**	55.80			
**R-Square**	0.8519			

Model Fitted: TY ~ PH + SN + RWC + PRO + H_2O_2 + POX + Tuber K^+/Na^+ +MI

Table S4. Regression coefficient, standard error, and significance of the prioritized traits for salinity stress tolerance

Dependent Variable	Variable entered	Regression coefficients (β_s)	Standard Error(SE)	t -Value	Pr(>|t|)
**TY (Tubers yield)**	Intercept	-1643.69	496.13	-3.31	0.002
	PH (**X1**)	12.47	1.26	9.86	0.000
	SN (**X2**)	40.82	9.78	4.17	0.000
	RWC (**X3**)	18.36	5.48	3.35	0.002
	PRO (**X4**)	-0.83	0.42	-1.99	0.053
	H_2O_2 (**X5**)	224.72	87.35	2.57	0.014
	POX(**X6**)	8.33	2.70	3.08	0.004
	Tuber K^+/Na^+ (**X7**)	37.02	12.01	3.08	0.004
	MI (**X8**)	-11.41	5.17	-2.20	0.033
**Model Fitted: TY~** -1643.69+ 12.47 X_1 **+** 40.82 X_2**+** 18.36 X_3**+ (**-0.83) X_4**+** 224.72 X_5**+** 8.33 X_6**+** 37.02 X_7**+ (**-11.41) X_8					
